# Development of Resistance Against Mefloquine Prophylaxis in Peace-Keeping Forces in the Central African Republic

**DOI:** 10.7759/cureus.8886

**Published:** 2020-06-28

**Authors:** Javaria Arshad Malik, Aqib Nadeem, Zahabia Khalid, Yasser Nadeem, Sehrish Zaffar, Amer H Siddiqui

**Affiliations:** 1 Pharmacology, Combined Military Hospital (CMH) Lahore Medical College, National University of Medical Sciences (NUMS), Lahore, PAK; 2 Aviation Medicine, Army Medical College, National University of Medical Sciences (NUMS), Islamabad, PAK; 3 Pharmacology, Postgraduate Medical Institute (PGMI), Lahore, PAK; 4 Ophthalmology, Combined Military Hospital (CMH) Lahore Medical College, National University of Medical Sciences (NUMS), Lahore, PAK

**Keywords:** prevalence, mefloquine resistance, anti-malarial therapy

## Abstract

Objective

To record the frequency of malaria-like symptoms in patients who have been given mefloquine as chemoprophylaxis and to determine the effectiveness of antimalarials against these symptoms.

Methodology

It was an observational study that took place at a United Nations Level 1 hospital, Kaga-Bandoro, Central African Republic. The total duration of the study was three months.

Patients presenting to the clinic with multiple symptoms despite chemoprophylaxis with mefloquine were assessed through a physical examination and screening test for malaria. Malaria treatment with a six-dose regimen of artemether (20 mg), along with lumefantrine (120 mg) two tablets BD for three days, was given after informed consent to those patients and post-treatment symptoms were observed and recorded.

Results

Out of 61 patients, 93% of them presented with body aches, 92% with headache, 52% with shivering, 44% with vertigo, 38% with fever, sweating, and nausea/vomiting, 18% with diarrhea, and 10% with pain in the abdomen.

Conclusion

It had been seen that patients presented with symptoms despite standard mefloquine prophylactic therapy, which were resolved with other antimalarial drugs. The presentation of the symptoms was also not classical.

## Introduction

Malaria is a mosquito-borne disease that presents with a wide range of symptoms. The prognosis of the disease may vary from full recovery to death depending upon the type of plasmodium species, host immune status, chemoprophylaxis use, and type of treatment administered. Although the tropical regions remain the hub of most malarial infection, with up to 500 million cases annually and a death toll of one million children each year, the international travelers are particularly at risk [1]. The true global incidence and prevalence of malaria is difficult to determine; the highest disease burden occurs in sub‐Saharan Africa where vital registration and disease notification systems are weak [2]. However, the latest World Health Organization (WHO) figures estimate 212 million new cases of malaria in 2015 leading to 429,000 deaths [3].

The high influx of travelers to malaria-endemic regions makes up to 125 million travelers prone to acquiring the disease hence there is a need for preventive measures [4]. It is approximated that 10 to 30 thousand travelers contact the disease [5]. Different methods for malaria prevention are vaccine, personal protection, and chemoprophylaxis. However, the use of the malaria vaccine is not on the near horizon, especially not for travelers, in spite of some new data that favors its use [6]. Personal protection, which includes barrier methods, mosquito repellants are an important tool but not satisfactory. Thus, chemoprophylaxis remains the major method to prevent malaria [7].

Mefloquine has been available for use in Europe since 1985 and the USA since 1990. Alongside atovaquone‐proguanil and doxycycline, it is considered standard chemoprophylaxis by many international health guidelines [8-10].

Mefloquine belongs to the arylamino acid group of antimalarial agents and is effective against all known strains of malaria that affect humans. The recommended dosage for mefloquine in antimalarial chemoprophylaxis is 5 mg/kg [11]. However, most international and national guidelines on malaria prevention recommend 250 mg per week as an effective dose in adult travelers [8,11-12].

It is natural for a clinician to rule out malaria as a possible diagnosis for patients who present with either vague or malaria-like symptoms but are taking mefloquine prophylaxis. The diagnosis can further be confused by a negative screening test. However, some studies indicate that even patients on mefloquine prophylaxis and a negative screening test may still be suffering from Malaria. The current study demonstrates the spectrum of symptoms with which patients on chemoprophylaxis presented and got successfully treated by an antimalarial regimen.

The purpose of this study was to detect and assess such patients clinically, provide them with standard malaria treatment, and then notify study results that patients do present with malaria despite mefloquine prophylaxis.

## Materials and methods

The study was conducted in a medical camp of the city of Kaga-Bandoro, of the Central African Republic. The details of the camp are not further described for reasons of operational security. The data was collected from March 1, 2017, to May 31, 2017, for a total period of three months. All patients presenting with malaria-like symptoms to the clinic during that time period were included in the study. All personnel in the medical camp were taking standard mefloquine prophylaxis, i.e., mefloquine 250 mg per week [12]. Patients not taking standard mefloquine prophylactic therapy were not included in the study.

Informed consent was taken from the patients and their symptoms were recorded in a self-developed questionnaire. Questions regarding malaria-like symptoms, such as fever, shivering, headache, sweating, body aches, vomiting, diarrhea, pain in the abdomen, and vertigo, were included in the questionnaire and assessed in the pre-treatment group. The screening test for malaria was also carried out. Patients showing any of these symptoms were given standard malaria treatment, i.e., the artemether (20 mg)-lumefantrine(120 mg) combination. After treatment, all malaria-like symptoms described above in all patients included in the study were re-assessed and entered in the questionnaire (Table 1).

**Table 1:** Malaria Patient Clinical Presentation Questionnaire

Malaria Patient Clinical Presentation Questionnaire										
Name										
Age										
Sex										
Case number										
Previous episodes of malaria										
Serial number	Symptoms		Days after treatment							
			1		2	3	4		5	6
1	Fever									
2	Shivering									
3	Headache									
4	Sweating									
5	Body pain									
6	Joint pain									
7	Nausea									
8	Vomiting									
9	Diarrhea									
10	Pain abdomen									
11	Vertigo									
12	Consciousness									
Serial number	Examination		Days after treatment							
			1		2	3	4		5	6
1	Temperature									
2	Pulse									
3	BP									
4	Palpable spleen									
5	Palpable liver									
6	ICI test (vector screen)									
Treatment given										
Remarks										

## Results

Out of 61, 23 patients (38%) presented with fever, 32 patients (52%) presented with shivering, 56 patients (92%) presented with headache, 23 patients (38%) presented with sweating, 57 patients (93%) presented with body aches, 23 patients (23%) presented with nausea/vomiting, 11 patients (18%) presented with diarrhea, six patients (10%) presented with pain in the abdomen, and 27 patients (44%) resented with vertigo. The vector screen was positive in only six patients (10%).

**Figure 1:**
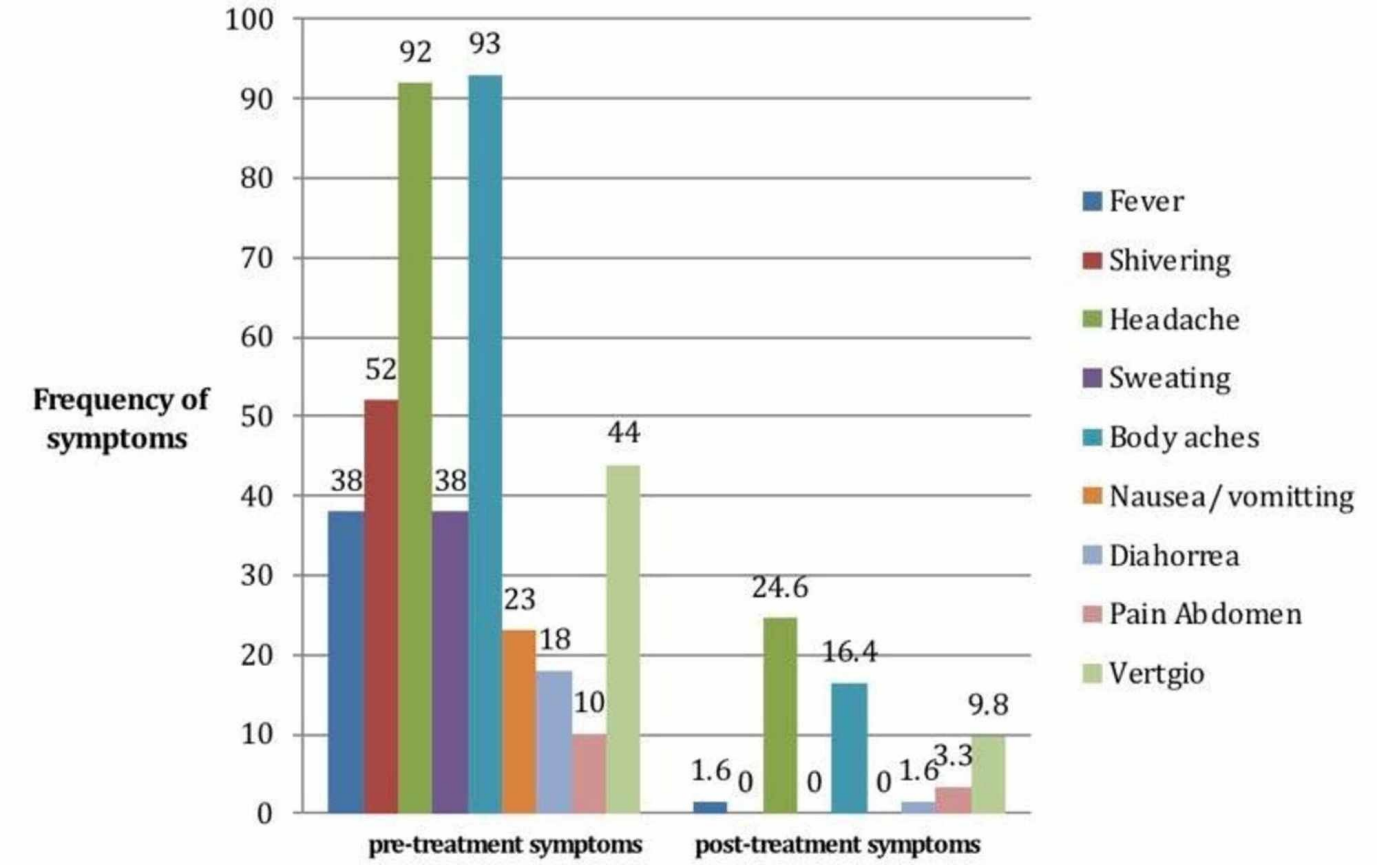
Frequency of patients with symptoms before and after standard malaria treatment (n=61)

Post-treatment, only 1.6% of patients had a fever, 25% had a headache, 16% had body aches, 1.6% had diarrhea, 3.3% patients had pain in the abdomen, and 10% patients had vertigo. Shivering, sweating, nausea, or vomiting were not observed after treatment (Figure 1).

According to the Wilcoxon signed-rank test, the p-value was significant for all the symptoms, signifying that the symptoms occurring in the patients were significantly resolved with the standard malaria treatment of the artemether-lumefantrine combination (Table 2). 

**Table 2:** Test statistics a. Wilcoxon signed-rank test; b. Based on positive ranks

Test Statistics^a^									
	post_fever - pre_fever	post_shivering - pre_shivering	post_headache - pre_headache	post_sweating - pre_sweating	post_bodyache - pre_bodyaches	post_NV - pre_NV	post_diarrhea - pre_diarrhea	post_painabd - pre_painabd	post_vertigo - pre_vertigo
Z	-4.690^b^	-5.657^b^	-6.403^b^	-4.796^b^	-6.856^b^	-4.796^b^	-3.162^b^	-2.000^b^	-4.583^b^
Asymp. Sig. (2-tailed)	.000	.000	.000	.000	.000	.000	.002	.046	.000

## Discussion

It is now realized that despite mefloquine prophylactic therapy, patients still developed symptoms that were resolved by other antimalarial drugs like the artemether-lumefantrine combination, which indicates the emergence of mefloquine resistance. This is critical information for a clinician in the field who may not consider the diagnosis of malaria based on chemoprophylaxis status. Such misdiagnosis can lead to greater damage to the patient or increase the severity of the disease [13-14].

Mefloquine resistance was occasionally reported first from the Thai-Cambodian border, then from other parts of Asia, and some from Africa and the Amazon region are also received. Mefloquine resistance in the area of the Thai-Cambodian and Thai-Burmese borders has reached 50%, so it is not permitted for use as prophylaxis in this particular region. Whereas, in all other regions, the drug can be used because the resistance level presently is more subjective. However, safety and tolerability with Mefloquine is the main concern of travelers [15].

In 2013, a case study was published describing the failure of mefloquine prophylaxis in an oversize traveler to Mozambique, which indicates dose adjustment in oversized patients [16]. The mechanism of failure of prophylactic therapy in the obese may be related to the volume of distribution and the systemic clearance of the individual, which are likely altered by body fat thus impacting the pharmacokinetic properties of the drug [17]. Since none of the participants in the current study were overweight, the mechanism of failure of prophylaxis is most likely attributable to some degree of drug resistance.

Mefloquine therapy had presented with varied symptoms like headache, vertigo, nausea, vomiting, and diarrhea as opposed to high-grade intermittent fever along with rigors and chills, mefloquine does seem to alter the course of the disease and decrease the severity of the outcome. No patient in the study developed any serious complications of malaria such as cerebral malaria, organ failure, etc. Although individuals not taking mefloquine prophylaxis were not part of the current study, yet symptoms reported by participants of the study were significantly milder and recovered much faster in comparison to known cases of malaria in the region. The advent of drug-resistant malaria is a great menace to tropical communities [18-19]. Early detection of the failure of malaria treatment regimens is important for regulating public health procedures in disease-endemic areas. Commonly, such decision-making depends on the results of clinical studies that evaluate the therapeutic efficacy of antimalarials, sometimes reinforced by in-vitro sensitivity testing.

Most of the symptoms with which patients had presented were resolved after standard malaria treatment, showing that the symptoms were, in fact, caused by malaria and not any other disease. Additional research needs to be done to comment on the causes of mefloquine resistance. The limitation of our study was the detection of malarial strains using microscopy and blood analysis of pre-treatment plasma mefloquine concentration. Therefore, this study does not comment on the prevalence of resistant strains of plasmodium. These data can be used in the future for further analysis. On the basis of results, it is recommended that patients presented with symptoms despite standard mefloquine prophylactic therapy. The presentation of the symptoms was also either mild or vague hence could not be classified as classical malaria symptoms. Therefore, clinicians in a malaria-endemic region must be armed with this knowledge to reduce the possibility of misdiagnosis and start the intervention as early as possible.

## Conclusions

Based on the results of this study, it can be concluded that mefloquine resistance has emerged in certain areas of the Central African Republic.
